# Prognostic value of changes in high-sensitivity cardiac troponin T beyond biological variation in stable outpatients with cardiovascular disease: a validation study

**DOI:** 10.1007/s00392-021-01952-6

**Published:** 2021-10-25

**Authors:** Moritz Biener, Evangelos Giannitsis, Katharina Hogrefe, Matthias Mueller-Hennessen, Hanna Fröhlich, Hugo A. Katus, Norbert Frey, Lutz Frankenstein, Tobias Täger

**Affiliations:** Zentrum für Innere Medizin, Klinik für Kardiologie, Angiologie und Pneumologie, Universitätsklinikum Heidelberg, Heidelberg, Germany

**Keywords:** High-sensitivity Troponin T, Biological variation, Outpatients, Outcomes, Cardiovascular disease

## Abstract

**Objective:**

To evaluate the prognostic implications of longitudinal long-term changes beyond the biological variation of high-sensitivity cardiac troponin T (hs-cTnT) in outpatients with stable or asymptomatic cardiovascular disease (CV) and to assess possible differences in the prognostic value while using reference change value (RCV) and minimal important differences (MID) as metric for biological variation.

**Methods:**

Hs-cTnT was measured at index visit and after 12 months in outpatients presenting for routine follow-up. The prognostic relevance of a concentration change of hs-cTnT values exceeding the biological variation defined by RCV and MID of a healthy population within the next 12 months following the stable initial period was determined regarding three endpoints: all-cause mortality (EP1), a composite of all-cause mortality, non-fatal myocardial infarction and stroke (EP2), and a composite of all-cause mortality, non-fatal myocardial infarction, stroke, hospitalization for acute coronary syndrome (ACS) or decompensated heart failure, and planned and unplanned percutaneous coronary interventions (PCI, EP3).

**Results:**

Change in hs-cTnT values exceeding the biovariability defined by MID but not by RCV discriminated a group with a higher cardiovascular risk profile. Changes within MID were associated with uneventful course (NPV 91.6–99.7%) while changes exceeding MID were associated with a higher occurrence of all endpoints within the next 365 days indicating a 5.5-fold increased risk for EP 1 (*p* = 0.041) a 2.4-fold increased risk for EP 2 (*p* = 0.049) and a 1.9-fold increased risk for EP 3 (*p* < 0.0001).

**Conclusions:**

In stable outpatients MID calculated from hs-cTnT changes measured 365 ± 120 days apart are helpful to predict an uneventful clinical course.

**Clinical trials identifier:**

NCT01954303.

**Graphic abstract:**

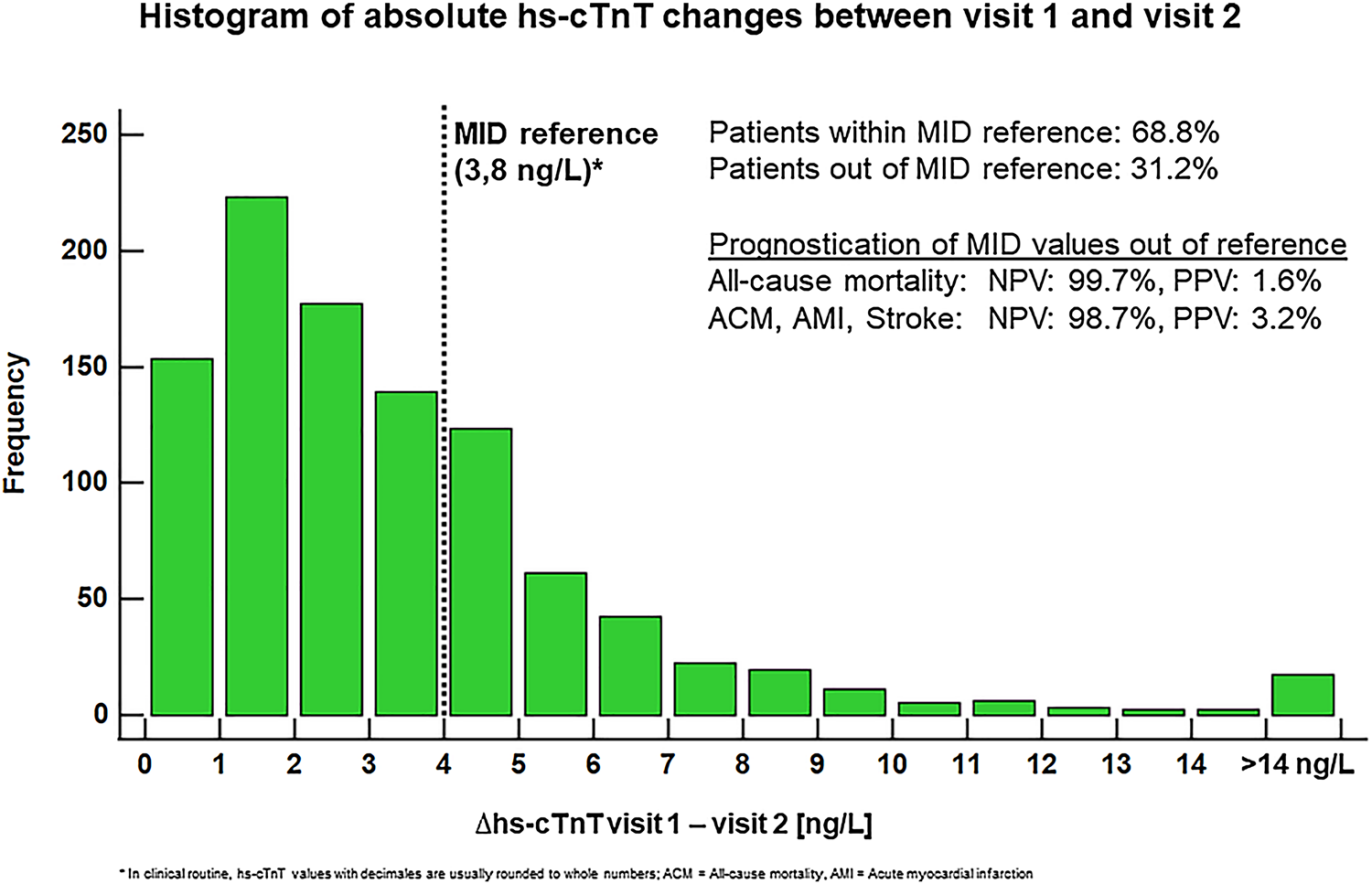

**Supplementary Information:**

The online version contains supplementary material available at 10.1007/s00392-021-01952-6.

## Introduction

Cardiovascular (CV) disease remains worldwide leading cause of morbidity and mortality [1]. Accordingly, patients suffering from CV conditions are at high risk for complications including heart failure, acute myocardial infarction, stroke or death [2]. In Germany, patients with stable CV diseases are seen every 6–12 months in an outpatient department or by a resident cardiologist where non-invasive procedures such as ECG or echocardiography are performed to evaluate a progression of the disease [3]. Those patients may benefit from a more intense diagnostic workup and/or shorter follow-up intervals, or non-invasive or invasive coronary imaging or function testing. While international guidelines recommend risk scores for the evaluation of patients with an acute coronary syndrome (ACS) such as the GRACE Score for secondary prevention [4], and the Framingham-, PROCAM- or the ESC-SCORE for primary prevention [5], no clinical risk score is available for the stratification of patients with pre-existing cardiovascular diseases for secondary prevention.

Cardiac troponins are suggested as preferred biomarkers for the identification of myocardial infarction and indicate myocyte injury [4]. Due to their high sensitivity, they not only allow to identify non-ST-segment elevation acute myocardial infarction (NSTEMI), but also indicate myocardial injury due to non-coronary and non-cardiac diseases [6, 7]. In addition to their value as diagnostic biomarker, cardiac troponins can also be used as prognostic biomarkers since they have proved to be indicative of future cardiovascular events including death irrespective of the underlying release mechanism [8–10]. Trials that evaluated the prognostic usability of cardiac troponins used pre-defined cutoffs (e.g., quartiles or the 99th percentile of a reference population) measured at a single timepoint. However, the consideration of only one troponin value disregards diurnal fluctuations and long-term variation of troponin over days, weeks or months around a homeostatic equilibrium. Changes that exceed biological variation may be particularly indicative of future cardiovascular events.

We recently published data on the long-term biological variation of high-sensitivity troponin T (hs-cTnT) in a population with stable cardiovascular disease free of endpoints [11]. Using reference change values (RCV) and minimal important difference (MID), we found a baseline-dependent biological variation within 12 months that was consistent among important subgroups.

Thus, the aim of this study was to validate the prognostic value of hs-cTnT kinetic changes exceeding the biological variation prospectively in a population of outpatients with CV disease free of endpoints and to evaluate whether there is a difference in the findings concerning changes beyond MID and RCV.

## Methods

### Study population

Patients were recruited from the HAK outpatient clinic at the Department of Cardiology, University Hospital Heidelberg, Germany. This population comprises stable outpatients with and without CAD or CV disease including previous ACS or coronary interventions, peripheral artery disease, hypertensive heart disease, valvular heart disease, chronic heart failure, venous thromboembolism and arrhythmias including atrial fibrillation. All individuals presenting between July 1, 2010 and December 31, 2016 were screened for eligibility.

Main inclusion criteria were a second presentation within 365 ± 120 days after index visit and available hs-cTnT values at both presentations. Only patients with a stable clinical course between index visit and follow-up visit were enrolled. Stable disease was defined by the absence of acute cardiac decompensation, recent ACS or coronary intervention since the previous visit. In addition, patients after heart transplant were not considered for the statistical analysis.

The diagnostic workup comprised a 12-lead-ECG, stress testing (ECG, echocardiography or MRI), carotid ultrasonography, CT coronary angiography, cardiac MRI, pulmonary function testing as well as Holter ECG and 24 h blood pressure recording. The selection of specific tests and the number of diagnostic tests was left at the discretion of the treating physician. A typical check-up contained a resting ECG, a 2D transthoracic echocardiogram, a carotid scan, and a stress ECG or stress imaging taking into consideration criteria that disqualified for stress ECG including factors that may confound interpretation of ECG or limited exercise capacity. Furthermore, laboratory testing including hs-cTnT, blood count, clinical chemistry and coagulation was performed. Patients received regular follow-up visits with follow-up examination and medical tests. All patients received a follow-up of at least 12 months beyond the uneventful 12-month period that had qualified for enrollment. Follow-up was executed using medical history, questionnaire or telephone contact. Ethical approval was waived due to the retrospective character of the study. All data were processed in an anonymized way.

### Definition of endpoints

We defined three prognostic endpoints (EP): (1) all-cause mortality (ACM, EP1); (2) a composite of ACM, non-fatal AMI and stroke (EP2) and (3) a composite of ACM, non-fatal AMI, stroke, hospitalization for ACS as well as planned and unplanned coronary interventions (EP3). ACM was defined as death from any cause including CV and non-CV conditions. AMI was defined according to the European Society of Cardiology fourth universal definition of myocardial infarction including ST-segment elevation (STEMI) and non-ST-segment elevation myocardial infarction (NSTEMI) [12]. Ischemic stroke was defined according to the updated definition of stroke for the twenty-first century of the American Heart Association/American Stroke Association [13]. ACS was defined according to the European Society of Cardiology Guidelines (ESC) on the Management of ACS in patients presenting without persistent ST-segment elevation [4]. A planned coronary intervention was defined as scheduled coronary angiography or percutaneous coronary intervention (PCI) whereas all unscheduled and emergency interventions were defined as unplanned.

### Laboratory measurements

Troponin measurements were performed during routine presentations. Cardiac troponin was measured in plasma on a COBAS E411 using the hs-cTnT assay by Roche Diagnostics. The limit of blank and limit of detection have been determined as 3 ng/l and 5 ng/l, respectively [14]. The 10% CV was determined at 13 ng/l in 100 measurements in the authors’ laboratory. The interassay CV was 8% at 10 ng/l and 2.5% at 100 ng/l. The intraassay CV was 5% at 10 ng/l and 1% at 100 ng/l. The hs-cTnT assays were not affected by a lot-to-lot variation that occurred in 2009 and 2010 [15].

### Statistical analysis

Variables were tested for normal distribution using the D’Agostino–Pearson test and were presented either as means ± standard deviation, or as medians with 25th and 75th percentiles. Categorial variables were compared using Chi^2^ or Fisher’s exact test. Continuous variables were compared using either Student *t* test for parametric or Mann–Whitney *U* test for nonparametric variables. Alternatively, we used ANOVA after logarithmic transformation of the data. If the ANOVA test was found positive (*p* < 0.05), then the Student–Newman–Keuls test for pairwise comparison of subgroups was applied. All tests were 2-tailed and a *p* value < 0.05 was considered statistically significant.

Biological variation over 12 months as published elsewhere was used as reference for test positive individuals [11]. Absolute changes of hs-cTnT over the period of 12 months were compared to the MID and relative changes to RCV, respectively. Changes exceeding biovariability defined by RCV and in a second comparison by MID of the respective group were classified as test positive and compared to true positives for every EP in a two by two table using Stata 16. Whereas only EP positive patients with an endpoint within 12 months were counted as true positives.

The prognostic performance was tested using three methods. First, we compared the prediction of all endpoints by RCV versus MID using area under the curve, and tested whether RCV and MID added prognostic information beyond hs-cTnT using logistic regression. Second, we compared the predictive value of RCV and MID using logrank with Kaplan–Meier survival analysis. Third, we compared the Chi^2^-values from Cox regression analysis.

## Results

### Baseline characteristics

**Fig. 1:**
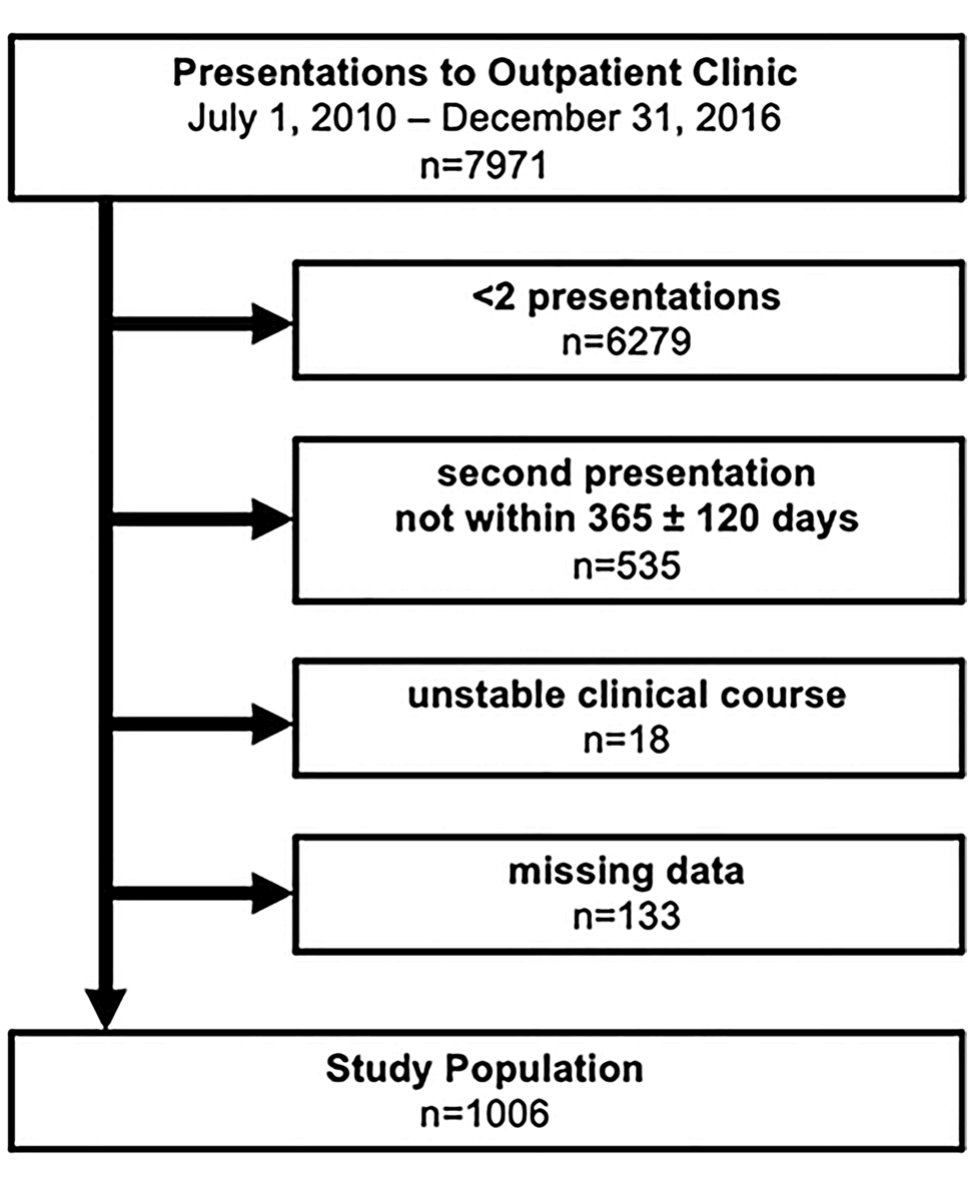
Study population

A total of 7971 patients were screened for eligibility. The final study cohort comprised 1006 patients, with two hs-cTnT measurements in the detectable concentration range, collected within the pre-specified observation period of 365 ± 120 days (Fig. 1). Baseline characteristics are displayed in Table 1. MID values out of reference were observed in 314 (31.2%) of the patients whereas RCV values exceeding the reference range were found in 230 (22.9%) individuals. Patients who had MID values exceeding the upper limit of the previously derived normal were older compared to those within reference whereas patients with RCV values exceeding reference values were younger than those with values within the reference range. Furthermore, patients with MID values out of reference were more likely to be male compared to those within reference, but no differences were observed in patients within or out of RCV reference values. The cardiovascular risk factor diabetes mellitus was found more often in patients with MID values out of the reference value whereas arterial hypertension and dyslipidemia were observed less often in patients with RCV values out of the reference range. A medical history of coronary intervention, aorto-coronary bypass graft, heart failure and chronic kidney disease was found more often in patients exceeding the MID reference value. In patients exceeding the RCV reference a medical history of coronary interventions was documented less often than in patients within the reference range. Higher hs-cTnT values at index and follow-up visits, higher NT-proBNP values and lower eGFR values were observed in patients with MID values out of the reference range whereas lower hs-cTnT values and a higher eGFR was found in patients exceeding the RCV reference. Days between index and follow-up visits did not differ in patients within or out of MID and RCV reference values.

**Table 1:** Baseline characteristics

	All	Minimal important difference		Reference change value	
		Within reference	Out of reference	Within reference	Out of reference
*n*	1006	692 (68.8)	314 (31.2)	776 (77.1)	230 (22.9)
Age (years)	69 (61–74)	68 (60–73)	71 (63–76)***	70 (62–75)	66 (58–73)***
Male gender (*n*, %)	761 (76)	509 (74)	252 (80)*	592 (76)	169 (74)
Risk factors (*n*, %)					
Arterial hypertension	853 (84)	586 (85)	267 (85)	674 (87)	179 (78)**
Diabetes mellitus	188 (19)	115 (17)	73 (23)*	152 (20)	36 (16)
Dyslipidemia	845 (84)	576 (83)	269 (86)	665 (86)	180 (78)**
Smoking	32 (3)	20 (3)	12 (4)	25 (3)	7 (3)
Medical history (*n*, %)					
Acute myocardial infarction	234 (23)	158 (23)	76 (24)	191 (25)	43 (19)
Coronary intervention	670 (67)	440 (64)	230 (73)**	534 (69)	136 (59)**
Aorto-coronary bypass graft	132 (13)	76 (11)	56 (18)**	106 (14)	26 (11)
Heart failure	172 (17)	102 (15)	70 (22)**	135 (17)	37 (16)
Cardiomyopathy	79 (8)	56 (8)	23 (7)	64 (8)	15 (7)
Stroke	49 (5)	29 (4)	20 (6)	36 (5)	13 (6)
Chronic kidney disease	102 (10)	52 (8)	50 (16)***	82 (11)	20 (9)
Malignancy	117 (12)	73 (11)	44 (14)	94 (12)	23 (10)
Clinical chemistry					
1. hs-cTnT (ng/l)	9 (5–13)	8 (5–11)	11 (7–18)***	9 (6–14)	7 (4–10)**
2. hs-cTnT (ng/l)	9 (6–14)	8 (5–11)	14 (8–21)***	10 (6–15)	7 (4–9)
MID^a^ (ng/l)	3.8	3.8	3.8	3.8	3.8
RCV^a^ (%)	44.2	44.2	44.2	44.2	44.2
RCVabs^b^ (ng/l)	4 (2–6)	4 (2–5)	5 (3–8)	4 (3–6)	2 (2–4)
NT-proBNP (ng/l)	162 (67–425)	149 (70–370)	208 (91–651)***	172 (82–450)	128 (68–312)
Follow-up					
Days between visits	365 (336–391)	365 (335–389)	365 (338–394)	364 (334–388)	367 (347–397)
Follow-up (days)	2030 (1960–2074)	2043 (1982–2113)	1951 (1873–2067)	2002 (1935–2050)	2158 (1970–2253)*
Endpoints within 1 year					
Endpoint 1	7 (1)	2 (0)	5 (2)***	6 (1)	1 (0)
Endpoint 2	19 (2)	9 (1)	10 (3)*	17 (2)	2 (1)
Endpoint 3	106 (11)	58 (8)	48 (15)***	88 (11)	18 (8)

*hs-cTnT* high-sensitivity troponin T, *eGFR* estimated glomerular filtration rate, *NT-proBNP* N-terminal brain natriuretic peptide

*p* value for the comparison of MID and RCV values within or out of reference *< 0.05, **< 0.01, ***< 0.001

^a^Defined in Täger et al [11]

^b^Calculated absolute value of RCV using 1. Hs-cTnT value

### Prognostic performance of hs-cTnT biovariability

Median follow-up was 2030 (IQR: 1960–2074) days in the entire cohort (Table 1), while endpoints were censored after 1 year due to the evaluation of the prognostic performance for 1 year. Three patients died from cardiovascular causes (one with dilated cardiomyopathy, two with ischemic cardiomyopathy), one from stroke and one from sepsis. In two patients, the cause of death was unknown. All endpoints were more often observed in patients with MID out of reference whereas the occurrence of endpoints did not differ depending on RCV values exceeding the reference value. The performance of MID and RCV for prediction of pre-defined outcomes varied largely.

Table 2 displays sensitivities, specificities, positive predictive values and negative predictive values. Sensitivities for MID values out of the reference ranged from 45.3% (95% CI 35.6–55.2%) for EP 3 to 71.4% (95% CI 29.0–96.3%) for EP 1. With 14.3% (95% CI 0.4–57.9%) for EP 1 to 17.0% (95% CI 10.4–25.5%) for EP 3 sensitivities for RCV values exceeding the reference range were significantly lower. Specificities ranged from 69.1% (95% CI 66.1–71.9%) for EP 1 to 70.4% (95% CI 67.3–73.4%) for MID values out of the reference range and 76.4% (95% CI 73.5–79.2%) for EP 3 and 77.1% (95% CI 74.3–79.7%) for EP 1 and EP 2 for RCV values exceeding the reference. Positive predictive values were low for both MID (1.6% [95% CI 0.5–3.7%] to 15.3% [95% CI 11.5–19.8%]) and RCV values (0.4% [95% CI 0.0–2.4%] to 7.8% [95% CI 4.7–12.1%]) exceeding the reference range. Negative predictive values were high for MID (91.6% [95% CI 89.3–93.6%] to 99.7% [95% CI 99.0–100.0%]) and RCV values (88.7% [95% CI 86.2–90.8%] to 99.2% [95% CI 98.3–99.7%]) exceeding the reference range.

**Table 2:** Prognostic sensitivities, specificities, positive predictive values and negative predictive values for the prediction of different endpoints

%, 95% CI	Sensitivity	Specificity	PPV	NPV
Endpoint 1				
MID	71.4 (29.0–96.3)	69.1 (66.1–71.9)	1.6 (0.5–3.7)	99.7 (99.0–100.0)
RCV	14.3 (0.4–57.9)	77.1 (74.3–79.7)	0.4 (0.0–2.4)	99.2 (98.3–99.7)
Endpoint 2				
MID	52.6 (28.9–75.6)	69.2 (66.2–72.1)	3.2 (1.5–5.8)	98.7 (97.5–99.4)
RCV	14.3 (0.4–57.9)	77.1 (74.3–79.7)	0.4 (0.0–2.4)	99.2 (98.3–99.7)
Endpoint 3				
MID	45.3 (35.6–55.2)	70.4 (67.3–73.4)	15.3 (11.5–19.8)	91.6 (89.3–93.6)
RCV	17 (10.4–25.5)	76.4 (73.5–79.2)	7.8 (4.7–12.1)	88.7 (86.2–90.8)

Given the very high NPV, RCV and in particular MID within reference limits were able to rule-out outcome events within 12 months whereas specificity and PPV of MID and RCV were low.

**Fig. 2:**
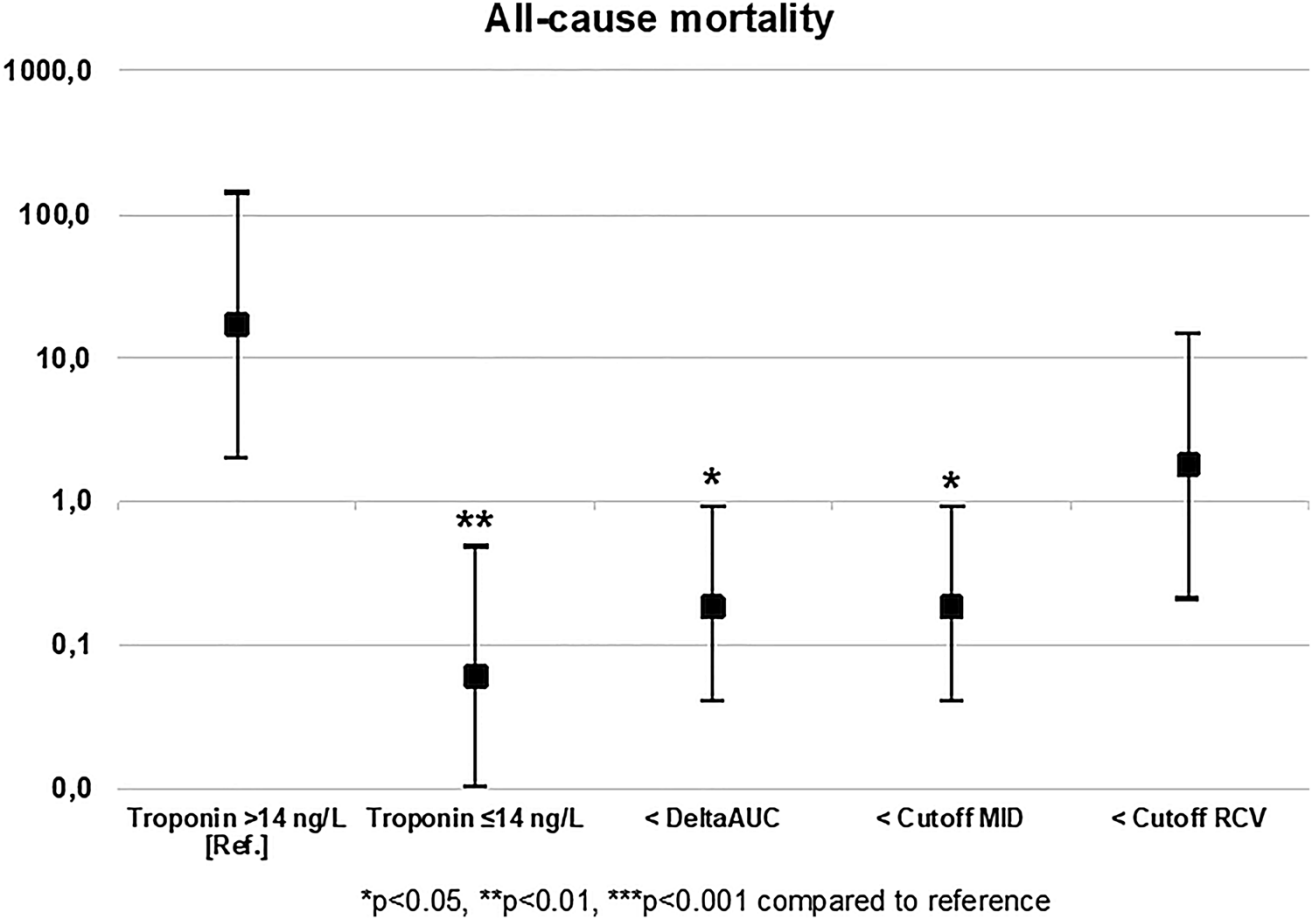
Hazard ratios for the endpoint. All-cause mortality depending on hs-cTnT ≤ 14 ng/l on first visit, hs-cTnT < ROC-optimized delta, and MID and ROC values within reference compared to the reference hs-cTnT > 14 ng/l at first visit

In a comparison of hazard ratios for the occurrence of EP 1, hs-cTnT values ≤ 14 ng/l (HR, 95 CI 0.06 [2.0–141.6], *p* = 0.0089), hs-cTnT delta values at first visit − second visit under the ROC-optimized cutoff (HR, 95 CI 0.18 [0.04–0.93], *p* = 0.0411) and MID values within the reference range (HR, 95 CI 0.18 [0.04–0.93], *p* = 0.0405) indicated a lower risk compared to the reference (i.e., hs-cTnT values > 14 ng/l on visit 1), whereas RCV values within the reference range (HR, 95 CI 1.78 [0.21–14.9], *p* = 0.5928) were not indicative of a lower risk compared to hs-cTnT values > 14 ng/l on visit 1 (Fig. 2). Hazard ratios for EP2 and EP3 can be found in the supplementary material (Figures S1 and S2). The predictive value of hs-cTnT for additional endpoints is displayed in Table S1. Hs-cTnT was also predictive for the combined endpoint re-admission for ACS and cardiovascular death as well es re-admission for ACS, but not for the endpoint cardiovascular death, that was observed in only three patients.

### Kaplan–Meier survival analysis and univariate Cox regression analysis

**Fig. 3:**
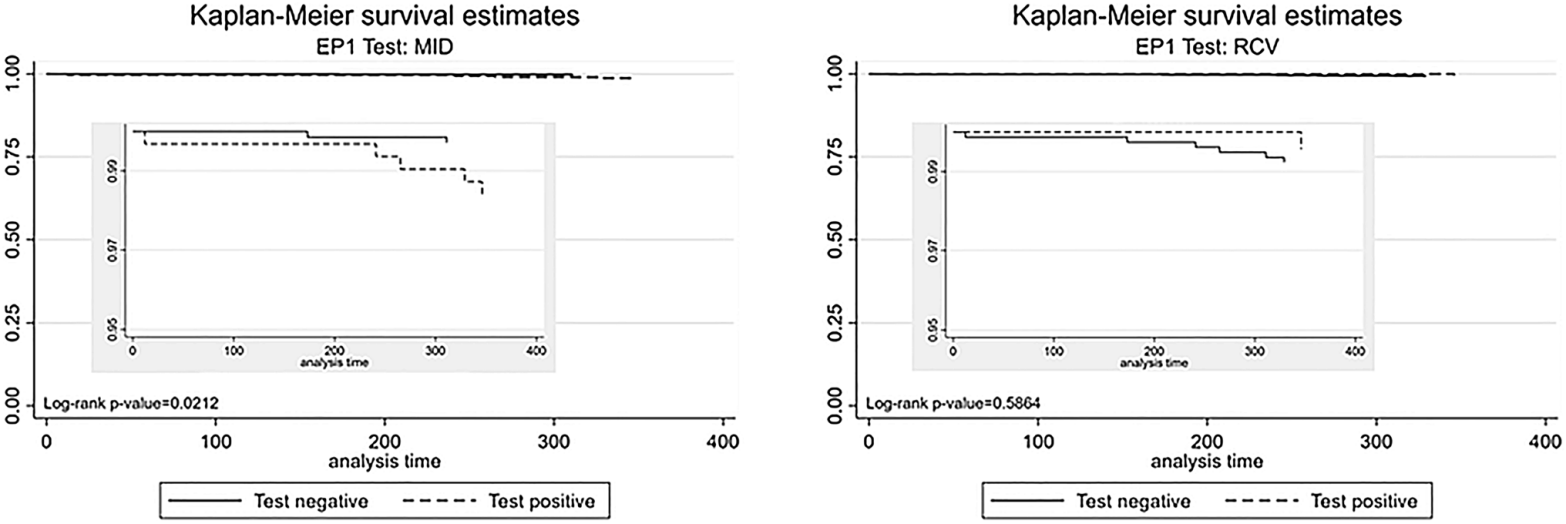
Kaplan–Meier survival curves for all-cause mortality depending on MID and RCV values within or out of reference

Kaplan–Meier curves for the occurrence of EP 1 depending on the change of hs-cTnT values exceeding biovariability defined by MID and RCV are displayed in Fig. 3*. *Use of MID at the pre-specified cutoff showed a significant difference for survival (Log-rank *p* value 0.0212) indicating a 5.5-fold higher risk for EP 1 (*p* = 0.041), whereas RCV at the pre-specified cutoff was not associated with a significant difference of survival (Log-rank *p* value 0.5864). Correspondingly, MID above reference also showed a significantly higher event rate for EP2 (Log-rank *p* value 0.0413, Figure S3) and EP3 (Log-rank *p* value 0.0005, Figure S4) indicating a 2.4-fold higher risk for EP 2 (*p* = 0.049) and a 1.9-fold higher risk for EP 3 (*p* < 0.0001), whereas RCV exceeding reference was not associated with difference in survival for EP2 (Log-rank *p* value 0.1972, Figure S3) or EP3 (Log-rank *p* value 0.1999, Figure S4) and had no predictive value for EP 2 (HR: 0.4, *p* = 0.213) and EP 3 (HR: 0.7, *p* = 0.202). Figure S5 displays interaction testing for the predictive value of MID and RCV depending on the comorbidities chronic kidney disease and atrial fibrillation. The prognostic value of MID differed according to the presence of CKD regarding EP 1 and CKD as well as atrial fibrillation regarding EP 2 and EP 3. RCV prognostic values only differed depending on the presence of CKD concerning EP 2. Hazard ratios for different hs-cTnT cutoffs as well as MID and RCV values exceeding the reference range depending on the presence of arterial hypertension, chronic kidney disease and atrial fibrillation are shown in Tables S2–S4.

### Multivariate Cox regression analysis

A cox proportional hazards regression model including age > 75 years, eGFR < 60 ml/min/1.73 m^2^ and NT-proBNP values at first visit is shown in Table 3. Neither hs-cTnT values > 14 ng/l at first and/or second visit, nor hs-cTnT values exceeding ROC-optimized delta values, or MID or RCV values out of the reference range remained predictive for EP1.

**Table 3:** Multivariate Cox proportional hazards regression model for the prediction of endpoint 1 considering hs-cTnT levels > 14 ng/l, MID and RCV values exceeding the reference value, age > 75 years, eGFR < 60 ml/min/1.73 m^2^ and NT-proBNP levels

Endpoint 1	Hazard ratio	95% CI low	95% CI high	*p* value
Any hs-cTnT > 14 ng/l	4.4	0.4	47.6	0.2257
Age > 75 years	3.1	0.6	17.7	0.1996
eGFR < 60 ml/min/1.73 m^2^	1.5	0.3	8.3	0.6214
NT-proBNP log10	**3.3**	**1.1**	**10.1**	**0.0356**
hs-cTnT at visit 1 > 14 ng/l	8.0	0.8	82.4	0.0794
Age > 75 years	3.0	0.5	16.2	0.2100
eGFR < 60 ml/min/1.73 m^2^	1.3	0.3	7.2	0.7312
NT-proBNP log10	2.9	1.0	8.8	0.0565
hs-cTnT at visit 2 > 14 ng/l	5.3	0.5	56.8	0.1709
Age > 75 years	2.9	0.5	16.6	0.2276
eGFR < 60 ml/min/1.73 m^2^	1.5	0.3	8.1	0.6414
NT-proBNP log10	3.2	1.1	9.7	0.0406
hs-cTnT deltaROC	2.7	0.5	14.8	0.2465
Age > 75 years	4.3	0.8	23.1	0.0907
eGFR < 60 ml/min/1.73 m^2^	1.8	0.3	10.0	0.4932
NT-proBNP log10	**3.7**	**1.3**	**10.7**	**0.0169**
MID exceeding reference	2.7	0.5	14.8	0.2457
Age > 75 years	4.3	0.8	23.0	0.0909
eGFR < 60 ml/min/1.73 m^2^	1.8	0.3	10.0	0.4937
NT-proBNP log10	**3.7**	**1.3**	**10.7**	**0.0169**
RCV exceeding reference	0.6	0.1	5.8	0.6528
Age > 75 years	4.1	0.7	23.0	0.1139
eGFR < 60 ml/min/1.73 m^2^	1.9	0.4	10.5	0.4582
NT-proBNP log10	**4.6**	**1.5**	**14.2**	**0.0088**

Bold values indicate statistical significance

## Discussion

The development of high-sensitivity troponin assays has allowed the detection of circulating troponin not only in patients with acute coronary syndromes, but also in stable cardiovascular disease and even in healthy individuals [8–10]. Moreover, an association of elevated troponin values with cardiovascular endpoints such as death, acute myocardial infarction or stroke has been reported in patients with acute coronary syndrome [16, 17] and stable cardiovascular disease [18, 19]. In the PEACE trial, hs-cTnT levels above the 99th percentile were associated with a 2.1-fold increased mortality rate in a population of patients with stable coronary heart disease and preserved left ventricular ejection fraction [18]. Furthermore, hs-cTnT values > 14 ng/l were associated with a doubling in the risk of AMI, stroke, heart failure and all-cause mortality in the BARI 2D trial, a cohort of patients with stable coronary heart disease and type 2 diabetes mellitus [20].

However, these trials evaluated the prognostic value of hs-cTnT considering a single value and used the 99th percentile of a reference population or tertiles or quartiles as a cutoff to indicate adverse outcomes [18–20]. Since patients with stable cardiovascular disease are typically seen at larger scheduled intervals, e.g., 6–12 months in an outpatient department or by an office cardiologist it is tempting to speculate that consideration of hs-cTnT long-term changes beyond biovariability better reflect a disease progression and might, therefore, be a more reliable risk indicator for cardiovascular events. In a recently published study with a population of stable outpatients with cardiovascular disease free of endpoints using MID and RCV, we had reported a hs-cTnT concentration-depending long-term biovariability that was consistent in important subgroups such as age, gender and renal function [11]. We, therefore, hypothesized that hs-cTnT changes larger than the biovariability of a population would indicate a higher risk for cardiovascular endpoints.

In stable outpatients with manifest cardiovascular disease, the use of a clinical score to predict the risk of future cardiovascular events is less well established than in primary prevention, and the overall acceptance of clinical scores outside clinical trials is low [21]. Therefore, the measurement of hs-cTnT which is established for the diagnosis of MI and specifically indicates myocardial injury has more recently attracted attention for its ability to predict outcomes in patients at high risk including T2 diabetes mellitus [20], stable CAD [19], chronic HF [22], but also in the general population [9]. In addition to that, our study group provided evidence on the prognostic role of elevated hs-cTnT in low-risk outpatients [23] and explored the role of concentration changes of hs-cTnT exceeding physiological biological variation [11]. In the present analysis, we validated the prognostic performance of the pre-specified cutoffs for RCV and MID that had been derived previously [11]. We report three important findings. First, changes within biovariability were associated with very high negative predictive values between 91.2 and 99.7% depending on the respective endpoint and thus ideal to predict an uneventful clinical course. In this regard, the NPV of MID was superior to RCV whereas the positive predictive value of MID and RCV were very low although RCV appeared to be less sensitive to confounding effects of atrial fibrillation and CKD. Second, changes of hs-cTnT beyond the biovariability defined by MID were associated with a higher cardiovascular risk profile and a higher all-cause mortality. However, when adjusted to elevated hs-cTnT > 99th percentile upper reference limit, or concentrations exceeding URL at presentation or follow-up, only NT-proBNP retained independent predictive power and hs-cTnT > URL demonstrated a trend for significance whereas values of MID and RCV were not independently predictive. In addition, MID demonstrated a significant interaction with the presence of atrial fibrillation or relevant CKD that was not observed for RCV regarding EP1 and EP2. However, atrial fibrillation, CKD and other confounders are known to increase hs-cTnT concentrations thus effecting the positive predictive value but should not have any impact within the low concentration range of MID.

In contrast, and to our surprise, changes beyond the biovariability defined by RCV were associated with a trend towards a more favorable risk profile and were not associated with mortality. This finding renders RCV less suitable for prediction of uneventful course. This discrepancy might—at least in part—be explained by the statistical method applied. Briefly, lacking a single indicator which can identify the exact moment of the progression of the disease, only the use of distribution-based approaches is feasible for the determination of biovariability in this cohort of stable patients with cardiovascular disease. MID and RCV are established metrices to illustrate biological variation. While MID utilizes absolute concentration changes, RCV is used as percent change, respectively. While the extend of relative changes depends on the magnitude of the initial hs-cTnT concentration, absolute changes do not vary for all patients. Implicating that using the RCV as cutoff every patient had a different value to be test positive, while using the MID as cutoff every patient was evaluated using the same hs-cTnT level. To the best of our knowledge no other MID or RCV have been reported that derived from a stable cohort. This would be necessary for the idea of a distribution-based approach which relies on the absence of any endpoints. Our neutral findings on the prognostic role of RCV is unexpected since Sandoval et al. had reported a 1.7-fold increased mortality risk after 3 months in stable hemodialysis patients exceeding RCVs [24]. In contrast to the stable low-risk population in our study, Sandoval had evaluated a hemodialysis cohort with a higher cardiovascular risk profile and a higher mortality rate (19% vs. 1% in our analysis) prohibiting a direct comparison. In addition, we adjusted for absolute hs-cTnT concentrations at baseline and after the first visit looking specifically at hs-cTnT exceeding the 99th percentile value. The latter was found to provide independent prognostic information beyond RCV. In contrast, hs-cTnT values almost always exceed the 99th percentile ULN in patients on hemodialysis which prohibits a meaningful comparison to our low-risk setting.

To the best of our knowledge, there is no study that evaluated risk stratification by MID in patients with stable cardiovascular disease so far. We, therefore, report for the first time a prognostic value for long-term hs-cTnT biovariability calculated by this metric. Both MID and RCV values had high negative predictive values between 88.7 and 99.7% so that patients with hs-cTnT values within the biovariability of a stable cohort seem to have a low risk to develop a cardiovascular endpoint. This is in keeping with previous studies that reported a low incidence of cardiovascular endpoints in patients with low hs-cTnT values [18–20, 23].

In accordance with studies on the prognostic value of hs-cTnT measured at a single time point from our group [16, 23] and others [9, 18] hs-cTnT levels exceeding the 99th percentile of a reference population were strongly indicative for mortality, whereas hs-cTnT values ≤ 14 ng/l as well as hs-cTnT kinetic changes < ROC-optimized cutoff and MID values within the reference range indicated a lower mortality risk. However, in a multivariate model considering other important predictors of cardiovascular endpoints neither hs-cTnT values > 14 ng/l or hs-cTnT delta changes nor MID nor RCV values exceeding the reference range were predictive for any endpoints. Interestingly, NT-proBNP values were predictive for EP1 in the multivariate analysis. Therefore, variation beyond the biovariability determined by MID seems not to be an independent risk predictor but possibly a metric to identify a group of higher risk.

In conclusion, our study indicates that biovariability in stable patients with cardiovascular disease should be measured using MID not RCV. Changes within biovariability as defined by MID and to a lower degree by RCV predict an uneventful course within at least 1 subsequent year. In addition, changes beyond this biovariability carry prognostic albeit not independent information for the prediction of mortality and combined endpoints in a low-risk outpatient population with cardiovascular disease. Even though the biovariability is not an independent risk predictor, it is an easy possibility to identify a patient group that might need a more excessive follow-up. Changes of 4 ng/l and more over 1 year merit a closer look at the patient.

## Limitations

One limitation of this study might be the single-center design. We, therefore, cannot exclude that our results may not be applied to other cohorts. Nevertheless, we recruited stable outpatients with a broad spectrum of cardiovascular disease and a typical clinical follow-up interval of 1 year. Furthermore, we documented a low rate of cardiovascular endpoints. This observation is characteristic for low-risk outpatient populations. Nonetheless, we were able to demonstrate a moderate prognostic value of hs-cTnT biovariability in this analysis. Due to the definition of the follow-up period at 365 ± 120 days, we are not able to apply our findings to longer or shorter follow-up intervals, but may provide information for the usual clinical follow-up period of ambulatory patients with stable cardiovascular disease.

## Supplementary Information

Below is the link to the electronic supplementary material.

Supplementary file1 (TIF 63 KB)

Supplementary file2 (TIF 63 KB)

Supplementary file3 (TIF 107 KB)

Supplementary file4 (TIF 111 KB)

Supplementary file5 (TIF 89 KB)

Supplementary file6 (DOCX 22 KB)
