# Supplementary figures and images for: Prognostic value of changes in high-sensitivity cardiac troponin T beyond biological variation in stable outpatients with cardiovascular disease: a validation study
Source: Clin Res Cardiol. 2021 Oct 25;111(3):333–42. doi: 10.1007/s00392-021-01952-6 (PMC8873128; doi:10.1007/s00392-021-01952-6)

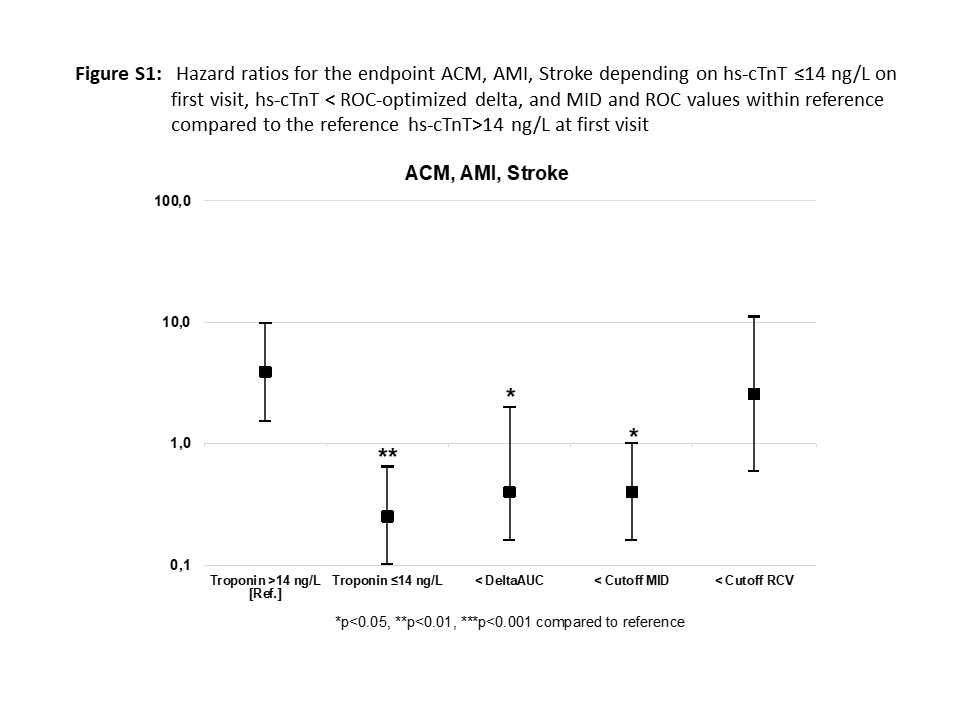

Supplement: Supplementary file 1 — Supplementary file1 (TIF 63 KB) [file 392_2021_1952_MOESM1_ESM.tif]

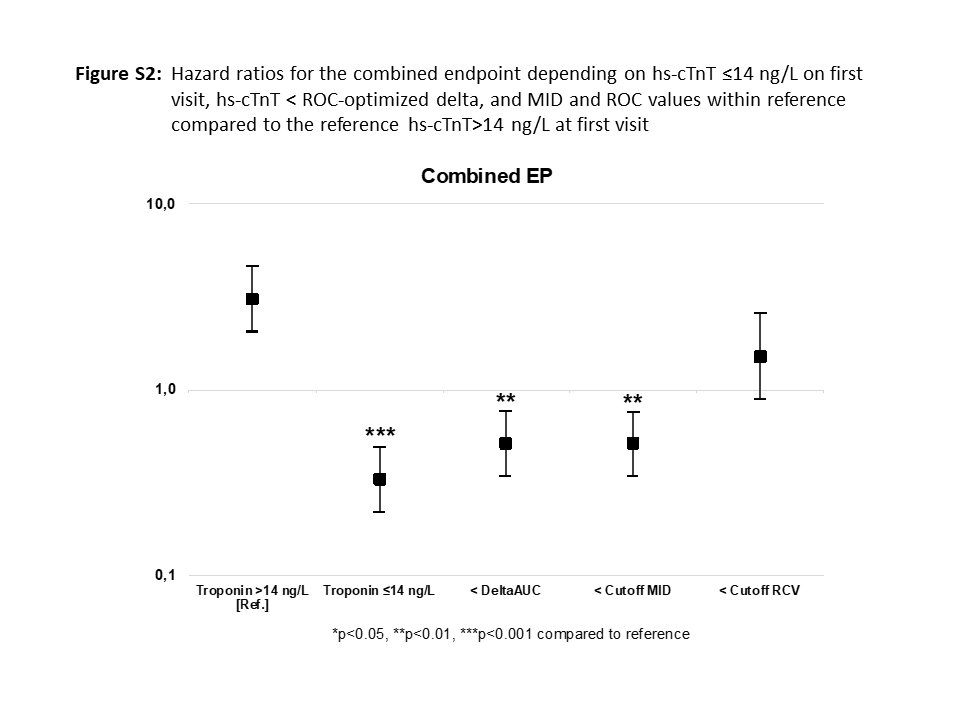

Supplement: Supplementary file 2 — Supplementary file2 (TIF 63 KB) [file 392_2021_1952_MOESM2_ESM.tif]

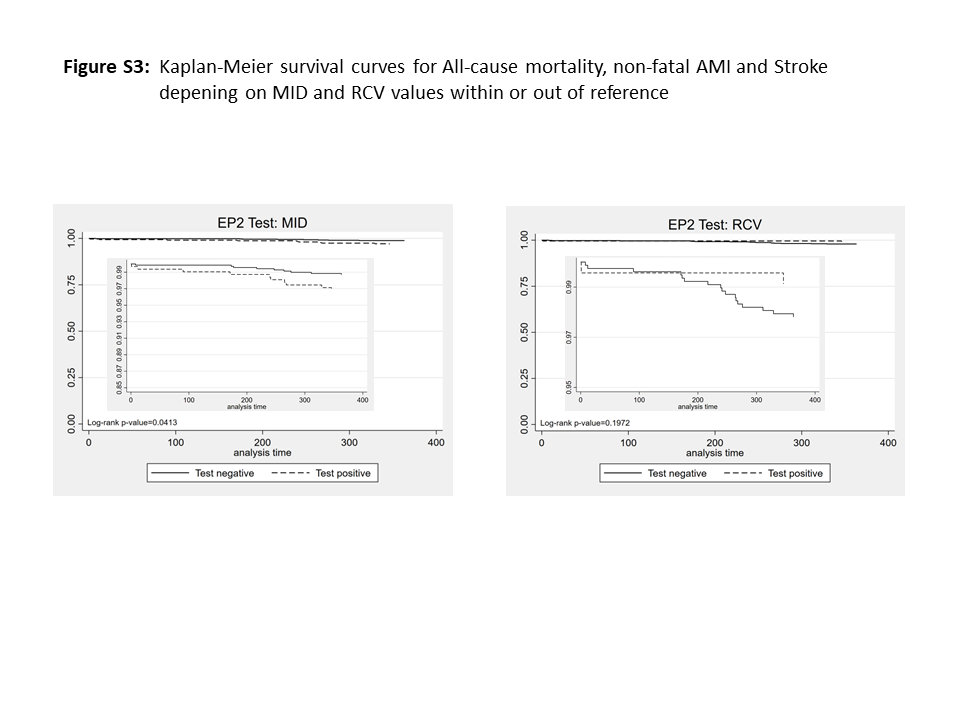

Supplement: Supplementary file 3 — Supplementary file3 (TIF 107 KB) [file 392_2021_1952_MOESM3_ESM.tif]

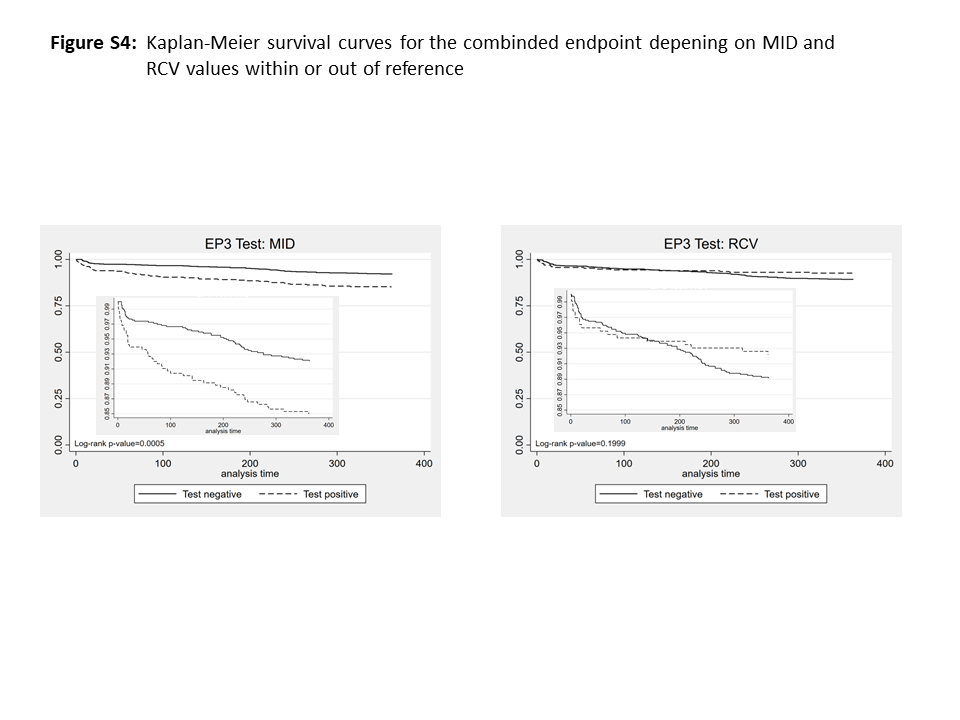

Supplement: Supplementary file 4 — Supplementary file4 (TIF 111 KB) [file 392_2021_1952_MOESM4_ESM.tif]

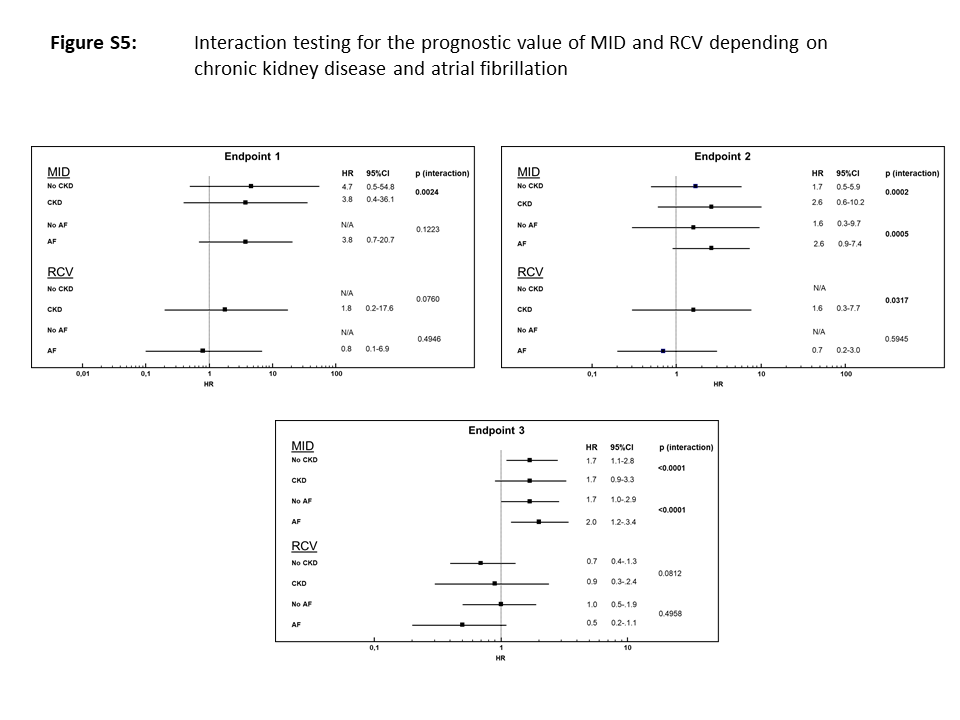

Supplement: Supplementary file 5 — Supplementary file5 (TIF 89 KB) [file 392_2021_1952_MOESM5_ESM.tif]
