# Supplementary material for: Prognostic value of changes in high-sensitivity cardiac troponin T beyond biological variation in stable outpatients with cardiovascular disease: a validation study
Source: Clin Res Cardiol. 2021 Oct 25;111(3):333–42. doi: 10.1007/s00392-021-01952-6 (PMC8873128; doi:10.1007/s00392-021-01952-6)
Supplement: Supplementary file 6 — Supplementary file6 (DOCX 22 KB) [file 392_2021_1952_MOESM6_ESM.docx]

**Table S1:** Hazard ratios of different hs-cTnT cutoffs for the endpoints rehospitalization for ACS and cardiovascular death

|  | **Hazard Ratio** | **95%CI low** | **95%CI high** | **p-value** |
| --- | --- | --- | --- | --- |
| **Rehospitalization for ACS/ Cardiovascular Death**  Any hs-cTnT >14 ng/L  Hs-cTnT at visit 1 >14 ng/L  Hs-cTnT at visit 2 >14 ng/L  Hs-cTnT > deltaROC  MID exceeding reference  RCV exceeding reference  **Rehospitalization for ACS**  Any hs-cTnT >14 ng/L  Hs-cTnT at visit 1 >14 ng/L  Hs-cTnT at visit 2 >14 ng/L  Hs-cTnT > deltaROC  MID exceeding reference  RCV exceeding reference  **Cardiovascular Death**  Any hs-cTnT >14 ng/L  Hs-cTnT at visit 1 >14 ng/L  Hs-cTnT at visit 2 >14 ng/L  Hs-cTnT > deltaROC  MID exceeding reference  RCV exceeding reference | **2.3**  **2.1**  **2.2**  1.6  1.3  0.7  **2.1**  **2.0**  **2.0**  1.5  1.3  0.7  2.8  4.0  3.2  N/A  11.1  0.7 | **1.4**  **1.2**  **1.3**  1.0  0.8  0.4  **1.3**  **1.2**  **1.2**  1.0  0.8  0.4  0.2  0.3  0.2  N/A  0.5  0.0 | **3.7**  **3.5**  **3.6**  2.5  2.0  1.3  **3.5**  **3.3**  **3.3**  2.5  2.1  1.4  44.5  65.0  50.8  N/A  231.5  14.1 | **0.0009**  **0.0058**  **0.0019**  0.0667  0.3101  0.2620  **0.0024**  **0.0124**  **0.0057**  0.0753  0.3674  0.3356  0.4711  0.3238  0.4158  N/A  0.1209  0.7977 |

**Table S2:** Hazard ratios for different hs-cTnT cutoffs and MID and RCV values exceeding the reference for endpoint 1 and endpoint 2 depending on a comorbidity of arterial hypertension

| HR, 95% CI | **Arterial Hypertension** | |
| --- | --- | --- |
|  | **Yes** | **No** |
| **Endpoint 1**  Any hs-cTnT >14 ng/L | 16.1 (3.1-82.5)  45.0 (7.4-272.9)  21.4 (4.0-115.0)  6.5 (1.3-32.0)  6.5 (1.3-32.3)  0.7 (0.1-4.1)  4.2 (1.6-11.3)  4.6 (1.6-13.9)  5.2 (1.9-14.4)  2.7 (1.0-7.3)  2.7 (1.0-7.3)  0.5 (0.2-1.6) | N/A  N/A  N/A  N/A  N/A  N/A  N/A  N/A  N/A  N/A  N/A  N/A |
| Hs-cTnT at visit 1 >14 ng/L |  |  |
| Hs-cTnT at visit 2 >14 ng/L |  |  |
| Hs-cTnT > deltaROC |  |  |
| MID exceeding reference |  |  |
| RCV exceeding reference |  |  |
|  |  |  |
| **Endpoint 2**  Any hs-cTnT >14 ng/L |  |  |
| Hs-cTnT at visit 1 >14 ng/L  Hs-cTnT at visit 2 >14 ng/L  Hs-cTnT > deltaROC  MID exceeding reference  RCV exceeding reference |  |  |

**Table S3:** Hazard ratios for different hs-cTnT cutoffs and MID and RCV values exceeding the reference for endpoint 1 and endpoint 2 depending on a comorbidity of chronic kidney disease

| HR, 95% CI | **Chronic kidney disease**  (eGFR rate <60 ml/min/1,73 m²) | |
| --- | --- | --- |
|  | **Yes** | **No** |
| **Endpoint 1**  Any hs-cTnT >14 ng/L | 1.8 (0.2-13.4)  2.6 (0.4-18.7)  2.1 (0.3-15.3)  3.5 (0.5-24.9)  3.5 (0.5-24.9)  2.1 (0.1-31.4)  1.3 (0.3-4.9)  1.2 (0.3-4.5)  1.5 (0.4-5.7)  2.5 (0.7-9.4)  2.5 (0.7-9.4)  1.7 (0.3-10.8) | N/A  N/A  N/A  6.3 (0.5-77.0)  6.4 (0.5-78.1)  N/A  6.5 (1.4-30.8)  8.6 (1.4-51.1)  8.9 (1.8-45.1)  1.7 (0.4-6.9)  1.7 (0.4-6.9)  N/A |
| Hs-cTnT at visit 1 >14 ng/L |  |  |
| Hs-cTnT at visit 2 >14 ng/L |  |  |
| Hs-cTnT > deltaROC |  |  |
| MID exceeding reference |  |  |
| RCV exceeding reference |  |  |
|  |  |  |
| **Endpoint 2**  Any hs-cTnT >14 ng/L |  |  |
| Hs-cTnT at visit 1 >14 ng/L  Hs-cTnT at visit 2 >14 ng/L  Hs-cTnT > deltaROC  MID exceeding reference  RCV exceeding reference |  |  |

**Table S4:** Hazard ratios for different hs-cTnT cutoffs and MID and RCV values exceeding the reference for endpoint 1 and endpoint 2 depending on a comorbidity of atrial fibrillation

| HR, 95% CI | **Atrial fibrillation** | |
| --- | --- | --- |
|  | **Yes** | **No** |
| **Endpoint 1**  Any hs-cTnT >14 ng/L | 6.5 (1.3-33.3)  13.6 (2.3-78.2)  7.9 (1.5-42.2)  4.2 (0.8-22.4)  4.2 (0.8-22.4)  0.8 (0.1-6.1)  3.0 (1.0-8.6)  2.7 (0.9-5.6)  3.5 (1.2-10.6)  2.8 (0.9-8.4)  2.8 (0.9-8.4)  0.7 (0.2-2.6) | N/A  N/A  N/A  N/A  N/A  N/A  3.9 (0.4-36.3)  8.5 (0.7-104.8)  5.1 (0.5-52.9)  1.7 (0.2-11.6)  1.7 (0.2-11.6)  N/A |
| Hs-cTnT at visit 1 >14 ng/L |  |  |
| Hs-cTnT at visit 2 >14 ng/L |  |  |
| Hs-cTnT > deltaROC |  |  |
| MID exceeding reference |  |  |
| RCV exceeding reference |  |  |
|  |  |  |
| **Endpoint 2**  Any hs-cTnT >14 ng/L |  |  |
| Hs-cTnT at visit 1 >14 ng/L  Hs-cTnT at visit 2 >14 ng/L  Hs-cTnT > deltaROC  MID exceeding reference  RCV exceeding reference |  |  |
